# Role of magnetic resonance imaging in the planning of breast cancer treatment strategies: comparison with conventional imaging techniques

**DOI:** 10.1590/0100-3984.2015.0124

**Published:** 2017

**Authors:** Luciana Karla Lira França, Almir Galvão Vieira Bitencourt, Hugo Lamartine Souza Paiva, Caroline Baptista Silva, Nara Pacheco Pereira, Jociana Paludo, Luciana Graziano, Camila Souza Guatelli, Juliana Alves de Souza, Elvira Ferreira Marques

**Affiliations:** 1 MD, Resident in Radiology and Diagnostic Imaging, A.C.Camargo Cancer Center, São Paulo, SP, Brazil; 2 PhD, Attending Physician, Imaging Department, A.C.Camargo Cancer Center, São Paulo, SP, Brazil; 3 MD, Attending Physician, Imaging Department, A.C.Camargo Cancer Center, São Paulo, SP, Brazil; 4 MD, Head of the Department of Breast Imaging, A.C.Camargo Cancer Center, São Paulo, SP, Brazil

**Keywords:** Breast neoplasms, Neoplasm staging, Magnetic resonance imaging

## Abstract

**Objective:**

To assess the role of magnetic resonance imaging (MRI) in the planning of breast cancer treatment strategies.

**Materials and Methods:**

The study included 160 women diagnosed with breast cancer, who underwent breast MRI for preoperative staging. Using Pearson's correlation coefficient (*r*), we compared the size of the primary tumor, as determined by MRI, by conventional imaging (mammography and ultrasound), and in the pathological examination (gold standard). The identification of lesions not identified in previous examinations was also evaluated, as was its influence on treatment planning.

**Results:**

The mean age of the patients was 52.2 years (range, 30–81 years), and the most common histological type was invasive ductal carcinoma (in 60.6% of the patients). In terms of the tumor size determined, MRI correlated better with the pathological examination than did mammography (*r* = 0.872 vs. 0.710) or ultrasound (*r* = 0.836 vs. 0.704). MRI identified additional lesions in 53 patients (33.1%), including malignant lesions in 20 (12.5%), which led to change in the therapeutic planning in 23 patients (14.4%).

**Conclusion:**

Breast MRI proved to be more accurate than conventional imaging in determining the dimensions of the main tumor and was able to identify lesions not identified by other methods evaluated, which altered the therapeutic planning in a significant proportion of cases.

## Abstract (pt)

**Objetivo:**

Avaliar o papel da ressonância magnética (RM) no planejamento terapêutico de pacientes com câncer de mama.

**Materiais e Métodos:**

Foram avaliadas 160 mulheres com diagnóstico de câncer de mama submetidas a RM para estadiamento préoperatório. O tamanho do tumor principal avaliado pela RM e pelos exames convencionais (mamografia e ultrassonografia) foi comparado com o exame anatomopatológico (padrão ouro), utilizando o coeficiente de correlação de Pearson (r). Foi avaliada, ainda, a presença de lesões adicionais não identificadas nos exames prévios e sua influência no planejamento terapêutico.

**Resultados:**

A idade média das pacientes foi 52,2 anos (variação: 30–81 anos) e o tipo histológico mais comum foi o carcinoma ductal invasivo (60,6%). A medida do tumor na RM teve melhor correlação com o tamanho no exame anatomopatológico, quando comparado com a mamografia (*r*: 0,872 × 0,710) e com a ultrassonografia (*r*: 0,836 × 0,704). A RM identificou lesões adicionais em 53 pacientes (33,1%), sendo 20 malignas (12,5%), e modificou o planejamento terapêutico em 23 pacientes (14,4%).

**Conclusão:**

A RM das mamas demonstrou ser mais acurada que os exames convencionais na avaliação das dimensões do tumor principal e foi capaz de identificar lesões adicionais não identificadas pelos outros métodos, que alteraram o planejamento terapêutico em um percentual importante dos casos.

## INTRODUCTION

Magnetic resonance imaging (MRI) has been increasingly used in the management of breast cancer. One of the main indications for breast MRI is for preoperative locoregional staging, given that the examination has high sensitivity for the assessment of tumor extent, as well as for the detection of multifocal and multicentric tumors^(1)^.

Studies have demonstrated that, in women diagnosed with breast cancer, the incidence of synchronous cancer in the ipsilateral breast can reach 27%, compared with 1–10% in the contralateral breast, and that the latter finding is associated with a worse prognosis^(2-7)^. The use of breast MRI for preoperative staging of the contralateral breast in patients diagnosed with breast cancer, is recommended by the American College of Radiology and the European Society of Breast Imaging^(8,9)^.

Questions persist regarding the role of breast MRI in patients who have been diagnosed with breast cancer and are eligible for conservative therapy. Various studies have shown that breast MRI is more accurate in the assessment of the tumor extent, as well as in the detection of multifocal and multicentric tumors, than are conventional examinations (mammography, ultrasound, and clinical examinations)^(10-16)^. Because of this greater accuracy, it is expected that breast MRI would increase the rates of complete resection, reduce the number of reoperations and improve the prognosis for such patients, although those effects have yet to be consistently demonstrated^(17-20)^.

The objective of this article was to assess the role of breast MRI in the preoperative staging of breast cancer patients, in the evaluation of the extent of the primary tumor, and in the investigation of additional lesions, as well as its effect on the planning of treatment strategies.

## MATERIALS AND METHODS

We assessed all patients who had been diagnosed with breast cancer diagnosis and underwent MRI for staging and treatment planning at our institution during the period from August 2012 to August 2014. The inclusion criteria were having received a histological diagnosis of breast cancer and having undergone breast MRI prior to any clinical or surgical treatment. The exclusion criteria were having undergone MRI at another institution and not having been followed after treatment. The final study sample comprised 160 women, with a mean age of 52.2 ± 11.5 years (range, 30–81 years). Among those women, mammography was indicated in 146 and ultrasound was indicated in 145. The size of the primary tumor assessed by MRI and by the conventional techniques (mammography and ultrasound) was compared with the results of the anatamopathological examination (gold standard). In addition, we assessed the presence of additional lesions (i.e., those not identified in the conventional examinations) and their influence on treatment planning.

After a review of the patient electronic records, a standard form was filled out, including MRI, mammography and ultrasound data, as well as histological results of the percutaneous biopsy and/or surgery. For the patients included in the study, a review of the breast MRI images was carried out by a radiologist with experience in breast imaging, with the aim of appropriately characterizing the lesions found. For the analysis of the mammography and ultrasound, the available reports of prior examinations in the medical charts were used. Histological data were obtained from the reports on file in the pathological anatomy department of the institution.

The patients who, after undergoing breast MRI, were referred for neoadjuvant chemotherapy, were not excluded, given that the study sought to assess the influence of MRI on treatment indications and the decision regarding the use of neoadjuvant chemotherapy can be influenced by the performance of this examination. In those cases, it was not possible to determine the correlation of the size of the lesions in the imaging examinations with the surgical specimen.

MRI images were obtained in a 1.5 T device (Signa HDxt; General Electric, Milwaukee, WI, USA), with a dedicated breast coil and patients in the prone position. Each examination consisted of images taken before and after the use of the paramagnetic contrast agent gadopentetate dimeglumine, at an infusion rate of 3 mL/s. Before the contrast administration, a three-dimensional (3D), pre-contrast T1-weighted gradient-echo sequence was obtained in the axial plane, at a slice thickness of 2.5 mm, and a T2/STIR, pre-contrast T2-weighted short-tau inversion-recovery sequence of both breasts was obtained in the sagittal plane, at a slice thickness of 4.0 mm. For the dynamic examination, five 3D, T1-weighted gradient-echo sequences, with fat suppression, were obtained in the axial plane. The first was obtained prior to the injection of the contrast, the second was obtained 20 s after injection of the contrast, and the others were obtained sequentially, over the following minutes. From these dynamic images, post-processing images are obtained, the precontrast image being subtracted from the post-contrast images to improve the visualization of the enhanced area. The last sequence consists of post-contrast, 3D, gradient-echo images of both breasts in the sagittal plane, with 1-mm thick slices and fat saturation.

The data obtained were stored in a database for statistical analysis with the SPSS Statistics software package, version 20.0 (IBM Corporation, Armonk, NY, USA). The descriptive analysis of the categorical variables consisted of the calculation of the absolute and relative frequencies. The numerical variables were described as mean and standard deviation (SD), with minimum and maximum values. For the assessment of the size of the primary tumor, the length of the long axis evaluated by the MRI and by the conventional imaging techniques, when available, was considered. We calculated Pearson's correlation coefficient (*r*) for each imaging method, using the pathological assessment as the gold standard. For that analysis, we considered only those patients for whom the dimensions of the primary tumor were described in the anatomopathological report, excluding those who received neoadjuvant chemotherapy. For an appropriate comparison between imaging methods (MRI versus mammography and MRI versus ultrasound), only those cases in which the size of the tumor had been noted in reports of prior mammography or ultrasound examinations were considered. Results for which the probability of a type I error was less than or equal to 5% (*p* ≤ 0.05) were considered statistically significant.

## RESULTS

The primary tumor presented on MRI as a mass in 121 cases (75.6%) and as non-mass enhancement in 39 (24.4%). The most common histological types were invasive ductal carcinoma (in 60.6%), invasive lobular carcinoma (in 13.8%), and ductal carcinoma *in situ* (in 7.5%). The mean length of the long axis of the primary tumor was 38.1 mm on MRI, 26.3 mm on mammography, 23.6 mm on ultrasound, and 26.8 mm in the anatomopathological examination.

Among the 146 patients who had previously undergone mammography, the most common findings for the primary tumor were mass in 73 (50.0%), microcalcifications in 31 (21.2%), architectural distortion in 18 (12.3%), focal asymmetry in 13 (8.9%), and absence of lesions in 11 (7.5%). Among the 145 patients who had previously undergone ultrasound, the most common findings were mass in 111 (76.6%), architectural distortion in 19 (13.1%), and absence of lesions in 15 (10.3%).

**Figure 1:**
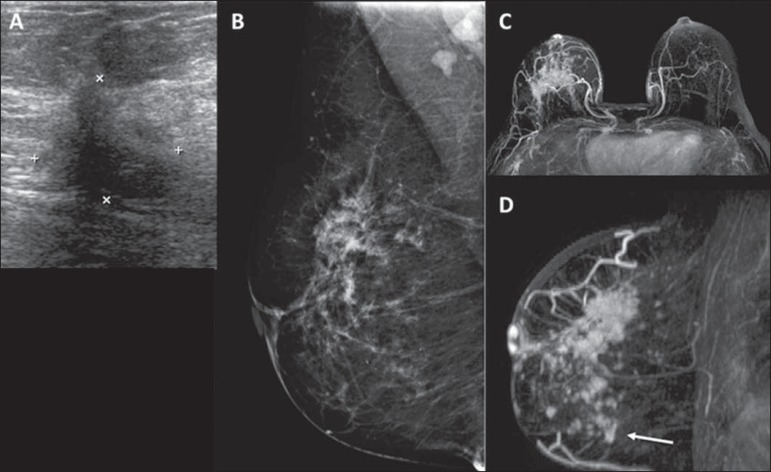
Ultrasound (**A**) showing an irregular hypoechoic mass in the upper outer quadrant of the right breast, which corresponds to the focal asymmetry in the mammogram (**B**). MRI showing a greater area of enhancement (**C,D**), extending to the lower quadrants (arrow).

Table 1 shows the length of the long axis of the primary tumor, as determined by mammography, ultrasound, MRI, and anatomopathology. In Table 2, the length of the long axis of the primary tumor assessed in the anatomopathological examination is correlated with that assessed by the various imaging techniques. The size of the tumor on MRI correlated better with the size determined in the anatomopathological examination that with the size determined by mammography (*r* = 0.872 × 0.710) and ultrasound (*r* = 0.836 × 0.704). Figure 1 illustrates the cases in which MRI contributed to better characterization of the extent of the primary tumor.

**Table 1:** Length of the long axis of the primary tumor on MRI, on mammography, on ultrasound, and in the anatomopathological examination.

	N	Minimum (mm)*	Maximum (mm)†	Median (mm)‡	Mean (mm)¶	Standard deviation
MRI	160	7	114	31.0	38.1	23.5
Mammography	80	5	80	23.5	26.3	15.6
Ultrasound	120	5	120	20.0	23.6	14.9
Anatomopathology	99	5	100	20.0	26.8	20.0

* Lowest value found;

† Highest value found;

‡ Value that separates the set into two equal groups;

¶ Sum of all values divided by the number of cases.

**Table 2:** Correlation between the length of the long axis of the primary tumor determined in the anatomopathological examination and that determined by the various imaging techniques (MRI, mammography, and ultrasound), evaluated by Pearson's correlation coefficient (r).

	N	Mean + standard deviation (mm)	*r*	*p*
Anatomopathology vs. MRI	101	Anatomopathology: 26.7 ± 19.9	0.730	< 0.001
		MRI: 33.2 ± 22.4		
Anatomopathology vs. MRI and mammography	52	Anatomopathology: 25.0 ± 18.1		
		MRI: 31.4 ± 21.0	0.872	< 0.001
		Mammography: 22.5 ± 14.9	0.710	< 0.001
Anatomopathology vs. MRI and ultrasound	79	Anatomopathology: 25.7 ± 19.3		
		MRI: 30.3 ± 19.6	0.836	< 0.001
		Ultrasound: 19.7 ± 11.3	0.704	< 0.001

**Figure 2:**
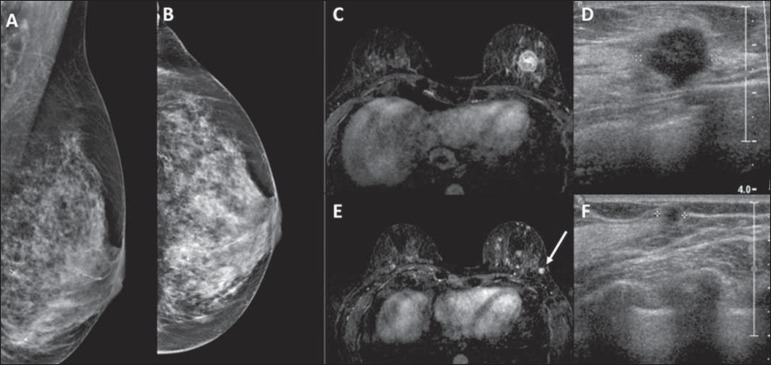
Patient with dense breasts on mammography (**A,B**). MRI and ultrasound (**C,D**) showing the primary tumor as an irregular mass in the left breast. The MRI scan also shows a small mass in the same breast (arrow in **E**), which was indentified in the second-look ultrasound examination (**F**). The biopsy confirmed invasive ductal carcinoma in both lesions.

MRI identified additional lesions in 53 patients (33.1%), the lesions being in the ipsilateral breast in 34 cases and in the contralateral breast in 19. Of those 53 lesions, 42 (79.2%) were masses and 11 (20.8%) were non-mass enhancements. The mean length of the long axis of the additional lesions was 12.6 ± 13.7 mm (range, 4–94 mm). Figure 2 illustrates the cases of additional lesions identified on MRI.

Of the additional lesions identified in MRI, 33 were submitted to histological study, 13 being benign and 20 being malignant (respectively corresponding to 8.1% and 12.5% of the sample as a whole). The material for the histological study was obtained by second-look ultrasound-guided or mammography-guided percutaneous biopsy (*n* = 16 and *n* = 1, respectively), or by surgical biopsy after preoperative ultrasound-guided or mammography-guided localization (*n* = 10 and *n* = 5, respectively). Among the malignant lesions, the most common histological types were invasive ductal carcinoma, in 9 (45.0%), invasive lobular carcinoma, in 2 (10.0%) and ductal carcinoma in situ, in 2 (10.0%). The additional lesions with low suspicion of malignancy that did not undergo histological study, by decision of the attending physician together with the patient, showed stability or regressed during the monitoring examinations, being monitored for a period of 12–24 months and being considered probably benign up until the end of the data collection period. Table 3 describes the number of additional malignant and benign lesions, by location and type of lesion.

**Table 3:** Additional lesions identified in the MRI, by location and type of lesion (*n* = 53).

	Result								
	Benign			Malignant			Total		
	N	%		%	N		N	%	*p*
Location									
Ipsilateral breast	21	61.8		13	38.2		34	100	1.000
Contralateral breast	12	63.2		7	36.8		19	100	
Type of lesion									
Mass	25	59.5		17	40.5		42	100	0.503
Non-mass enhancement	8	72.7		3	27.3		11	100	

The MRI findings prompted a change in the treatment planning in 23 patients with additional lesions (14.4% of the whole sample). An additional segmental resection was successfully undertaken in 12 cases, mastectomy was performed in ten cases, and the patient was referred for neoadjuvant chemotherapy in one case. Among the 12 patients in whom an additional resection was carried out, the additional resected lesion was found to be benign in 5 (50.0%). In the other cases in which there was an additional segmental resection, as well as in the cases in which the patient underwent mastectomy, the lesions were shown to be malignant (*n* = 17; 10.6% of the total).

## DISCUSSION

The results of the present study show that MRI correlated better with the size of the breast tumor found in the assessment of the surgical specimen than did mammography and ultrasound. In addition, breast MRI identified additional lesions in a significant proportion of the patients (33.1%), approximately a third being malignant, and prompted a change in the treatment planning in 14.4% of cases. Various authors have described the importance of breast MRI during preoperative staging, because it is more sensitive than conventional imaging techniques in the assessment of the tumor extent, even for ductal carcinoma *in situ* and invasive lobular carcinoma^(10,12,14,15,21-23)^. Furthermore, MRI has high sensitivity for the detection of multifocal, multicentric, and contralateral tumors. MRI can reportedly identify additional tumors in the ipsilateral breast in 15–27% of patients and in the contralateral breast in 1–10%. A preoperative assessment by MRI prompts a change in the treatment strategy in up to a third of breast cancer patients^(7,24)^. The foci of breast cancer identified using MRI are clinically significant in the majority of cases^(25)^. Second-look ultrasound, aimed at the assessment of these additional lesions identified using MRI, plays a fundamental role, because its permits the identification of the majority of the suspicious lesions, allowing a better assessment of the degree of their potential malignancy and the performance of the percutaneous biopsy or preoperative localization.

However, there is no consensus in the literature regarding the benefit to the patient provided by preoperative MRI. Whereas some studies have shown that the use of MRI reduces the rate of resections with positive margins, others have shown that, despite an increase in the number of mastectomies, explained by the greater number of malignant additional lesions identified by biopsy, there has been no reduction in the reoperation rate^(25-31)^. To date, there have been no prospective controlled studies demonstrating a reduction in recurrence or an increase in survival among breast cancer patients who undergo MRI for staging and treatment planning. In addition, we should investigate the number of additional surgical procedures prompted by MRI results and their impact in terms of morbidity and mortality over the medium and long term. However, it is necessary to point out the difficulty in demonstrating that a diagnostic method such as MRI can alter clinical or surgical outcomes, such as the rates of reoperation and mortality, due to the presence of diverse confounding factors related to the treatment itself, including different individual styles applied to the surgical techniques, possibly accountable for the variability of results. Therefore, we know only that MRI can offer additional information related to the extent of the disease, which will have an influence on the planning of the final treatment strategy.

Recently, a number of authors have shown that preoperative breast MRI can be more effective in specific subgroups. Young patients, patients with dense breasts, and patients diagnosed with invasive lobular carcinoma are among the subgroups that show the greatest benefit from MRI for treatment planning^(32)^. In addition, various molecular subtypes can influence the preoperative MRI assessment.

In recent years, neoadjuvant chemotherapy, which improves prognosis and achieves a complete pathological response, has been increasingly used in breast cancer patients. Breast MRI has been used ever more widely for the appropriate assessment of the response to treatment. In addition, various MRI parameters before the start of treatment have been used to predict treatment response and even survival in this patient population.

The results of the present study should be considered in view of its limitations. Because this was a retrospective study, it was not possible to assess the size of the tumor by all the conventional imaging techniques evaluated (mammography and ultrasound). Given that in many cases the mammography and ultrasound examinations were performed at other facilities, it was not possible to standardize the equipment employed or the review of the images, only the information included in the report of each examination being considered. It was also not possible to assess the size of the tumor in the surgical specimen in the patients who received neoadjuvant chemotherapy, the use of which continues to grow in Brazil. In addition, not all of the additional lesions identified on MRI were submitted to histological study, clinical-radiological monitoring being considered for the determination of benignity in these cases.

In conclusion, breast MRI proved to be more accurate than are conventional examinations in the assessment of the extent of the primary tumor and was capable of identifying additional lesions not identified by other methods, which altered the treatment planning in a significant proportion of the cases evaluated. Future prospective studies should be undertaken to assess the impact of these alterations on the morbidity and mortality over the medium and long term, as well as to define the real benefits of MRI for treatment planning in patients with breast cancer.
